# Retraction: An Integrated Approach to the Prediction of Chemotherapeutic Response in Patients with Breast Cancer

**DOI:** 10.1371/annotation/8f94e479-4161-43a0-a28c-4c0460bb89a4

**Published:** 2011-09-02

**Authors:** Kelly H. Salter, Chaitanya R. Acharya, Kelli S. Walters, Richard Redman, Ariel Anguiano, Katherine S. Garman, Carey K. Anders, Sayan Mukherjee, Holly K. Dressman, William T. Barry, Kelly P. Marcom, John Olson, Joseph R. Nevins, Anil Potti

The chemotherapy sensitivity predictions as reported in this PLoS One article were based on an approach as described by Potti et al. in Nature Medicine (1). Reexamination of the validation datasets used for the Nature Medicine study has revealed the presence of errors in the labeling of clinical response in some datasets (2). Re-analysis of the predictive accuracy with correctly labeled data has shown that in two instances the reported signatures do not predict the response of the validation samples to chemotherapy (2). The authors of the Nature Medicine paper have therefore decided to retract that paper (2). Since the PLoS One article is based on the approach reported in the Nature Medicine article, we have decided to retract the PLoS One article. We apologize to readers for any inconvenience caused by the publication of our article in PLoS One.

References:

1. Potti A, Dressman HK, Bild A, Riedel RF, Chan G, Sayer R, et al. Genomic signatures to guide the use of chemotherapeutics. Nat Med. 2006;12(11):1294-300.

2. Potti A, Dressman HK, Bild A, Riedel RF, Chan G, Sayer R, et al. Retraction: Genomic signatures to guide the use of chemotherapeutics. Nat Med. 2011; 17: 135. 

